# Association of CSF visinin-like protein 1 levels with cerebral glucose metabolism among older adults

**DOI:** 10.1371/journal.pone.0329386

**Published:** 2025-09-17

**Authors:** Zhaowei Wang, Sufeng Xiao, Meiping Wan, for the Alzheimer’s Disease Neuroimaging Initiative

**Affiliations:** 1 Department of Neurology, Qianjiang Central Hospital of Hubei Province, Hubei, China; 2 Department of Rehabilitation, Qianjiang Central Hospital of Hubei Province, Hubei, China; Banner Alzheimer's Institute, UNITED STATES OF AMERICA

## Abstract

**Background:**

CSF visinin-like protein 1 (VILIP-1) levels have exhibited potential utility as a marker of neuronal damage and are increased in Alzheimer’s disease (AD). The levels of CSF VILIP-1 have been associated with memory decline and hippocampal atrophy, while no studies have investigated the association between CSF VILIP-1 levels and cerebral glucose metabolism among older adults.

**Methods:**

Study participants had available baseline CSF VILIP-1 data and more than two assessments of 18-fluorodeoxyglucose positron emission tomography ([^18^F] FDG-PET) brain imaging. Linear mixed-effects models were used to examine the association between baseline CSF VILIP-1 levels and longitudinal changes in FDG-PET over time. Models were performed separately for the cognitively unimpaired (CU) and cognitively impaired (CI) participants.

**Results:**

Among CU older adults, higher CSF VILIP-1 levels were marginally associated with a faster reduction in cerebral glucose metabolism. In CI older adults, CSF VILIP-1 levels were significantly associated with a faster reduction in cerebral glucose metabolism.

**Conclusion:**

These results provide novel insights into the relationship between neuronal injury and cerebral glucose metabolism, highlighting the potential of CSF VILIP-1 as a biomarker for monitoring and predicting the progression of neurodegenerative processes.

## Introduction

Alzheimer’s disease (AD) is the most common neurodegenerative disease affecting older people [1]. Detecting and monitoring the disease early is crucial for managing it effectively and for possible treatments [2]. Positron emission tomography (PET) currently enables the visualization of AD pathophysiological alterations in living older people [3]. Cerebral glucose metabolism, as measured by [^18^F] fluorodeoxyglucose (FDG) PET, is a well-established marker of brain function and has been extensively used to evaluate the progression of neurodegenerative diseases [4–6]. Reduced cerebral glucose metabolism in specific brain regions, a hallmark of AD and other dementias, often precedes the onset of clinical symptoms [4,7]. This cerebral glucose hypometabolism is considered a neuroimaging marker of neurodegeneration in AD [2].

Cerebrospinal fluid (CSF) biomarkers have emerged as valuable tools in the diagnosis and prognosis of AD [8]. One such biomarker, Visinin-like Protein 1 (VILIP-1), is a calcium-sensing protein [9] that has been identified as a marker of neuronal injury [10,11]. Elevated levels of VILIP-1 have been associated with cognitive decline and neurodegeneration, highlighting its potential as a useful marker surrogate for neurodegeneration [12–20]. However, the relationship between CSF VILIP-1 levels and changes in cerebral glucose metabolism has not been explored among older adults.

In this study, we aimed to investigate the association between CSF VILIP-1 levels and changes in cerebral glucose metabolism, as measured by FDG-PET, among older adults. Understanding the relationship between CSF VILIP-1 levels and cerebral glucose metabolism can offer crucial insights into the underlying mechanisms of neurodegeneration. This study may contribute to the development of more accurate diagnostic tools and therapeutic strategies, ultimately enhancing the care and outcomes for older adults with cognitive impairment.

## Methods

### Participants

In this study, we included a total of 354 older adults classified as either cognitively unimpaired (CU), mild cognitive impairment (MCI), or mild AD dementia. Participants with MCI and mild AD dementia were categorized together as cognitively impaired (CI). Based on diagnostic criteria defined in the ADNI study, CU participants had a Mini-Mental State Examination (MMSE) [21] score ranging from 24 to 30 and a Clinical Dementia Rating (CDR) [22] score of 0. The criteria for MCI included an MMSE score between 24 and 30, a CDR of 0.5, a subjective memory complaint, and objective memory loss as measured by education-adjusted scores on the Wechsler Memory Scale Logical Memory II, without significant interference with daily life activities. The criteria for mild AD dementia included an MMSE score between 20 and 26, a CDR score of 0.5 or 1, and meeting the National Institute of Neurological and Communicative Disorders and Stroke–Alzheimer’s Disease and Related Disorders Association (NINCDS-ADRDA) [23] criteria for probable AD.

### Alzheimer’s Disease Neuroimaging Initiative (ADNI) study

This study used data from the ADNI study, which was launched in 2003. This study was designed to examine the usefulness of biological and neuroimaging markers for the prevention, detection, and treatment for AD. Detailed information on the ADNI study can be found online at http://adni.loni.usc.edu/ and has been described elsewhere [24]. Recruitment procedures for the ADNI study have been described previously [25] and can be found on the website (https://adni.loni.usc.edu/wp-content/uploads/2024/02/ADNI_General_Procedures_Manual.pdf). The current study selected ADNI participants who had available baseline CSF VILIP-1 data. In addition, all participants had more than two assessments of [^18^F] FDG-PET brain imaging available. All participants provided written informed consent. The ADNI study was approved by the institutional review board at each participating center.

### Assessment of cerebral glucose metabolism

Images were preprocessed at the University of Michigan using a standard procedure detailed on the following website (https://adni.loni.usc.edu/data-samples/adni-data/neuroimaging/pet/). The fully processed images were then downloaded from the ADNI database (http://adni.loni.ucla.edu/). ADNI investigators at the University of California, Berkeley, defined FDG-PET regions of interest (ROIs) based on a meta-analysis of studies that identified brain regions most frequently showing metabolic changes in AD or correlated with cognitive performance [4]. Five ROIs, labeled “MetaROIs,” were established and located in the bilateral posterior cingulate gyrus, bilateral angular gyri, and middle/inferior temporal gyrus. FDG standardized uptake value ratios (SUVRs), used in the current analysis, were defined using the average of the SUVRs from these five brain regions.

### Measurement of CSF VILIP-1 levels

The Neurogenomics and Informatics Center at Washington University used SomaLogic’s SomaScan platform to determine CSF VILIP-1 levels as part of their proteomic assessments. Each sample underwent hybridization normalization separately. Based on the signal-to-noise ratio in technical replicates and samples, aptamers were divided into three normalization groups: S1, S2, and S3. This separation was essential to avoid combining aptamers with varying protein signal intensities in subsequent normalization [26]. Following categorization, a median-based normalization method was utilized to correct for assay-related discrepancies, including protein concentration, pipetting errors, reagent concentration, and assay timing. CSF VILIP-1 levels are presented in relative fluorescence units (RFU), with the values being log-transformed before statistical analysis.

### Statistical analysis

Sample characteristics were presented as mean (SD) for continuous variables and n (percentage) for categorical variables. Both CSF VILIP-1 and FDG-PET met the assumption of normality. Two-sample t-tests were applied to compare differences in continuous variables between the two cognitive groups, and chi-square tests were used to compare differences in categorical variables. We conducted Pearson’s correlation analysis to examine the relationship between CSF VILIP-1 levels and cerebral glucose metabolism, as measured by FDG-PET. To examine the association between baseline CSF VILIP-1 levels and longitudinal changes in cerebral glucose metabolism over time, linear mixed-effects models were used separately for the CU and CI participants. Models were adjusted for age, gender, education, APOE4 status, amyloid status, and the interactions of these variables with follow-up years. Each model included a random intercept and a random slope. Specifically, a random intercept was included to account for individual baseline variability, and a random slope to capture subject-specific differences in the rate of change in FDG-PET over time. All statistical analyses were conducted using R software [27], and the significance level was set at p < 0.05.

## Results

### Comparison of sample characteristics between cognitive status

**Fig 1:**
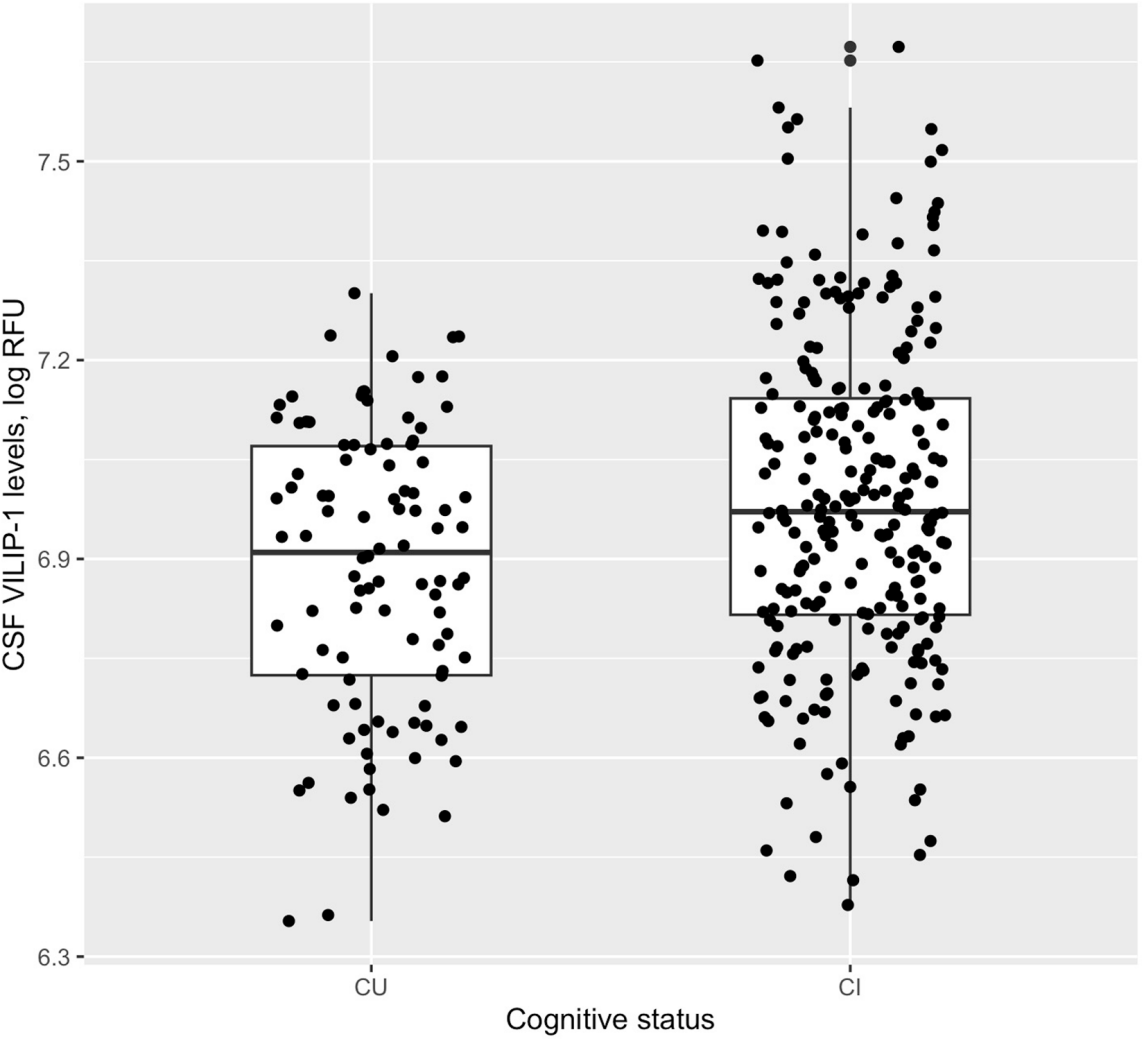
CSF VILIP-1 levels between cognitive status. CI participants had higher levels of CSF VILIP-1 relative to those of CU participants (t = −3.8406, p-value = 0.0001638). Abbreviations: CU: cognitively unimpaired; CI: cognitively impaired; VILIP-1: visinin-like protein 1; RFU: relative fluorescence units.

In the current study, a total of 354 participants were included, comprising 98 CU and 256 CI participants. As shown in Table 1, CU participants were older than CI participants. As expected, compared to CU participants, CI participants had higher percentages of APOE4 carriers and Aβ+ individuals, lower MMSE scores, and lower FDG SUVR levels. Consistent with previous studies, CI participants had higher levels of CSF VILIP-1 relative to those of CU participants (Table 1 and Fig 1). There were no significant differences in years of education or distribution of gender. Specifically, the CU group showed higher average FDG SUVRs compared to the CI group (Cohen’s d = 0.52, 95% CI [0.29, 0.76]). In addition, the CU group showed lower average CSF VILIP-1 levels compared to the CI group (Cohen’s d = −0.43, 95% CI [−0.66, −0.19]).

**Table 1:** Sample characteristics.

Characteristic	Overall, N = 354	CU, N = 98	CI, N = 256	p-value
Age, years	73 (7)	74 (6)	73 (8)	0.036
Education, years	16 (3)	16 (3)	16 (3)	0.078
Gender				0.7
Male	214 (60%)	61 (62%)	153 (60%)	
Female	140 (40%)	37 (38%)	103 (40%)	
APOE4 status				<0.001
APOE4-	196 (55%)	71 (72%)	125 (49%)	
APOE4+	158 (45%)	27 (28%)	131 (51%)	
MMSE	28 (2)	29 (1)	27 (2)	<0.001
Amyloid status				<0.001
Aβ-	131 (37%)	60 (61%)	71 (28%)	
Aβ+	223 (63%)	38 (39%)	185 (72%)	
FDG SUVRs	1.22 (0.14)	1.28 (0.11)	1.21 (0.14)	<0.001
CSF VILIP-1 levels, log RFU	6.96 (0.25)	6.89 (0.21)	6.99 (0.25)	<0.001

Notes: Variables are presented using Mean (SD) and n (%). Two-Sample t-tests and Pearson’s Chi-squared tests were used to compare variables between cognitive status.

Abbreviations: CU: cognitively unimpaired; CI: cognitively impaired; APOE4: apolipoprotein E ε4; Aβ: β-amyloid; FDG: fluorodeoxyglucose; SUVRs: standardized uptake value ratios; VILIP-1: visinin-like protein 1; RFU: relative fluorescence units.

### Cross-sectional relationship between CSF VILIP-1 levels and cerebral glucose metabolism

**Fig 2:**
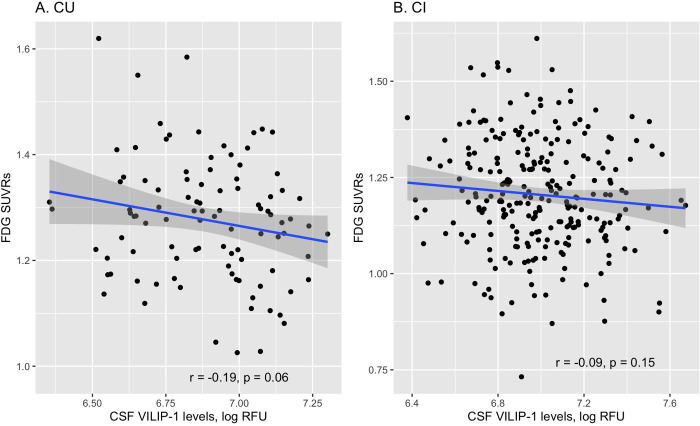
Cross-sectional relationship between CSF VILIP-1 levels and cerebral glucose metabolism in the CU and CI participants. Abbreviations: CU: cognitively unimpaired; CI: cognitively impaired; FDG: fluorodeoxyglucose; SUVRs: standardized uptake value ratios; VILIP-1: visinin-like protein 1; RFU: relative fluorescence units.

To examine the relationship between CSF VILIP-1 levels and cerebral glucose metabolism among older adults, Pearson’s correlation analyses were performed in the overall sample and separately for the CU and CI groups. In the overall sample, CSF VILIP-1 levels were negatively correlated with cerebral glucose metabolism (r = −0.15, p = 0.005). In the CU group, however, CSF VILIP-1 levels were marginally correlated with cerebral glucose metabolism (r = −0.19, p = 0.06, Fig 2A). In the CI group, CSF VILIP-1 levels were not correlated with cerebral glucose metabolism (r = −0.09, p = 0.15, Fig 2B).

### Association of baseline CSF VILIP-1 levels with longitudinal changes in cerebral glucose metabolism

**Fig 3:**
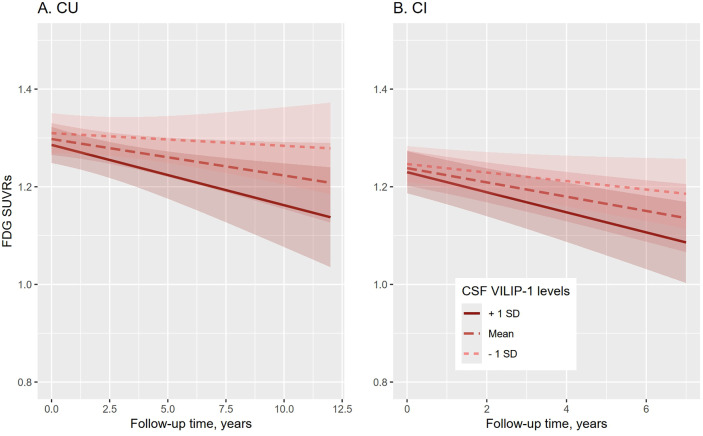
Association of CSF VILIP-1 levels with changes in cerebral glucose metabolism across cognitive status. Abbreviations: CU: cognitively unimpaired; CI: cognitively impaired; FDG: fluorodeoxyglucose; SUVRs: standardized uptake value ratios; VILIP-1: visinin-like protein 1.

To investigate whether CSF VILIP-1 levels are associated with changes in cerebral glucose metabolism, linear mixed-effects models were conducted separately for the CU and CI participants. Results of the CU and CI models are summarized in Table 2. Each model included the main effects of CSF VILIP-1 and covariates. For the sake of brevity, only the interaction terms with time are presented in Table 2. In the CU model, after adjusting for covariates, higher CSF VILIP-1 levels were marginally associated with a faster reduction in cerebral glucose metabolism (coefficient: −0.023; 95% CI: −0.046 to 0.000; p = 0.050; Table 2 and Fig 3A). In the CI model, higher CSF VILIP-1 levels were significantly associated with a faster reduction in cerebral glucose metabolism (coefficient: −0.023; 95% CI: −0.039 to −0.008; p = 0.003; Fig 3B).

**Table 2:** Summary of linear mixed-effect models.

	CU model Coefficient [95% CIs]	P values	CI model Coefficient [95% CIs]	P values
Age × years	0.000 [−0.001, 0.001]	0.736	0.000 [−0.001, 0.000]	0.362
Female gender × years	0.002 [−0.008, 0.013]	0.631	−0.002 [−0.009, 0.006]	0.663
Education × years	0.000 [−0.002, 0.001]	0.685	0.000 [−0.002, 0.001]	0.642
APOE4 status × years	0.007 [−0.004, 0.018]	0.220	−0.001 [−0.010, 0.008]	0.773
Amyloid status × years	−0.021 [−0.032, −0.011]	<0.001	−0.015 [−0.024, −0.005]	0.003
CSF VILIP-1 × years	−0.023 [−0.046, 0.000]	0.050	−0.023 [−0.039, −0.008]	0.003

Notes: Each model included the main effects of CSF VILIP-1 and covariates. For the sake of brevity, only the interaction terms with time are presented.

Abbreviations: CU: cognitively unimpaired; CI: cognitively impaired; CIs: confidence intervals; APOE4: apolipoprotein E ε4; VILIP-1: visinin-like protein 1.

## Discussion

This study examined the association of CSF VILIP-1 levels with cerebral glucose metabolism among older adults with and without cognitive impairment. Consistent with previous studies, CI participants exhibited higher levels of CSF VILIP-1 than CU participants. Higher CSF VILIP-1 levels were marginally associated with a faster reduction in cerebral glucose metabolism among CU older adults. Additionally, we found that CSF VILIP-1 levels were significantly associated with a faster reduction in cerebral glucose metabolism among CI older adults. These results provided novel insights into the relationship between neuronal injury and cerebral glucose metabolism, underscoring the potential of CSF VILIP-1 as a biomarker for monitoring and predicting the progression of neurodegenerative processes.

Consistent with previous studies [12,20], we observed that CI participants exhibited higher levels of CSF VILIP-1 compared to CU participants. This finding is in line with the role of VILIP-1 as a marker of neuronal injury [11]. Increased levels of CSF VILIP-1 are associated with faster rates of cognitive decline [12] and hippocampal atrophy [16]. The higher levels of VILIP-1 in CI participants suggest a greater degree of neuronal damage or dysfunction in this group, which is a hallmark of various neurodegenerative conditions, including AD [20].

Our study revealed that higher CSF VILIP-1 levels were marginally associated with a faster reduction in cerebral glucose metabolism over time among CU older adults. This suggests that even in the absence of clinically detectable cognitive impairment, subclinical neuronal injury may be associated with early metabolic changes in the brain. This finding is consistent with previous studies showing that alterations in cerebral glucose metabolism can precede the onset of cognitive symptoms [7]. Additionally, we found that CSF VILIP-1 levels were significantly associated with a faster reduction in cerebral glucose metabolism among CI older adults. This association underscores the potential of VILIP-1 as a robust biomarker for monitoring the progression of neurodegeneration [28]. The significant correlation between CSF VILIP-1 and cerebral glucose metabolism in CI participants indicates that VILIP-1 not only reflects the extent of neuronal injury but also predicts the rate of metabolic decline over time, which is a crucial manifestation in the clinical course of AD and other dementias [6]. The relationship between VILIP-1 and cerebral glucose metabolism provides important mechanistic insights. Neuronal injury, as reflected by elevated VILIP-1, may lead to damage to synapses [29] and, consequently, a decrease in glucose utilization. This interplay between neuronal health and metabolic function highlights the complex and dynamic nature of neurodegenerative processes. Understanding this relationship could have significant implications for the development of therapeutic strategies aimed at preserving neuronal integrity and maintaining cerebral metabolism.

Several limitations should be acknowledged. First, the sample size, particularly for the CU group, was relatively small, which may limit the generalizability of our findings. Future studies with larger and more diverse cohorts are needed to confirm and extend these findings. Second, the observational nature of the study design limits our ability to establish causality. Third, while we adjusted for several covariates, there may be other unmeasured confounders, such as comorbidities or lifestyle factors, that could influence the results. Finally, the specificity of VILIP-1 as a biomarker for different types of neurodegenerative diseases needs further investigation, as it may reflect neuronal injury in a variety of conditions.

In conclusion, our findings demonstrate that CSF VILIP-1 levels are associated with a faster reduction in cerebral glucose metabolism, particularly among CI older adults. This association supports the potential of VILIP-1 as a valuable biomarker for monitoring and predicting the progression of neurodegenerative diseases.

## Supporting information

S1 Text Detailed Acknowledgments for the ADNI Database. (DOCX)

S2 File The full membership list of the ADNI. (PDF)
