# Supplementary material for: Association of CSF visinin-like protein 1 levels with cerebral glucose metabolism among older adults
Source: PLoS One. 2025 Sep 17;20(9):e0329386. doi: 10.1371/journal.pone.0329386 (PMC12443256; doi:10.1371/journal.pone.0329386)
Supplement: S1 Text — (DOCX) [file pone.0329386.s001.docx]

S1 Text. Detailed Acknowledgments for the ADNI Database.

Data used in the preparation of this article were obtained from the Alzheimer′s Disease Neuroimaging Initiative (ADNI) database (adni.loni.usc.edu). As such, the investigators within the ADNI contributed to the design and implementation of ADNI and/or provided data but did not participate in the analysis or writing of this report. A complete listing of ADNI investigators can be found at: <http://adni.loni.usc.edu/wp-content/uploads/how_to_apply/ADNI_Acknowledgement_List.pdf>. The leader of the ADNI project is Prof. Michael Weiner (email: [michael.weiner@ucsf.edu](mailto:michael.weiner@ucsf.edu)).

Data collection and sharing for this project was funded by the Alzheimer's Disease Neuroimaging Initiative (ADNI) (National Institutes of Health Grant U01 AG024904) and DOD ADNI (Department of Defense award number W81XWH-12-2-0012). ADNI is funded by the National Institute on Aging, the National Institute of Biomedical Imaging and Bioengineering, and through generous contributions from the following: AbbVie, Alzheimer’s Association; Alzheimer’s Drug Discovery Foundation; Araclon Biotech; BioClinica, Inc.; Biogen; Bristol-Myers Squibb Company; CereSpir, Inc.; Cogstate; Eisai Inc.; Elan Pharmaceuticals, Inc.; Eli Lilly and Company; EuroImmun; F. Hoffmann-La Roche Ltd and its affiliated company Genentech, Inc.; Fujirebio; GE Healthcare; IXICO Ltd.; Janssen Alzheimer Immunotherapy Research & Development, LLC.; Johnson & Johnson Pharmaceutical Research & Development LLC.; Lumosity; Lundbeck; Merck & Co., Inc.; Meso Scale Diagnostics, LLC.; NeuroRx Research; Neurotrack Technologies; Novartis Pharmaceuticals Corporation; Pfizer Inc.; Piramal Imaging; Servier; Takeda Pharmaceutical Company; and Transition Therapeutics. The Canadian Institutes of Health Research is providing funds to support ADNI clinical sites in Canada. Private sector contributions are facilitated by the Foundation for the National Institutes of Health (www.fnih.org). The grantee organization is the Northern California Institute for Research and Education, and the study is coordinated by the Alzheimer’s Therapeutic Research Institute at the University of Southern California. ADNI data are disseminated by the Laboratory for Neuro Imaging at the University of Southern California.

﻿Data used in preparation of this article were generated by the Neurogenomics and Informatics Center at Washington University (https://neurogenomics.wustl.edu/). As such, the investigators within the NGI provided data but did not participate in analysis or writing of this report. A complete listing of NGI investigators can be found at: https://neurogenomics.wustl.edu/about-us/leadership/]

CSF Somalogic (7K) and Metabolomics (Metabolon HD4) data generation and QC was supported by grants from the National Institutes of Health (RF1AG074007 (PI: Yun Ju Sung), R01AG044546 (PI: Carlos Cruchaga), P01AG003991(PI: John Morris and Carlos Cruchaga), RF1AG053303 (PI: Carlos Cruchaga), RF1AG058501 (PI: Carlos Cruchaga), and U01AG058922 (PI: Carlos Cruchaga)), and the Chan Zuckerberg Initiative (CZI), and the Alzheimer’s Association Zenith Fellows Award (ZEN-22-848604, awarded to Carlos Cruchaga).

The recruitment and clinical characterization of research participants at Washington University were supported by NIH P30AG066444 (PI: John Morris and Carlos Cruchaga), P01AG03991(PI: John Morris and Carlos Cruchaga), and P01AG026276 (PI: John Morris and Carlos Cruchaga).

This work was supported by access to equipment made possible by the Hope Center for Neurological Disorders, the Neurogenomics and Informatics Center (NGI: https://neurogenomics.wustl.edu/)and the Departments of Neurology and Psychiatry at Washington University School of Medicine.
